# The Contradiction of a Benign Metastatic Meningioma: A Case Report

**DOI:** 10.7759/cureus.94462

**Published:** 2025-10-13

**Authors:** Amber Ahmed-Issap, Kajan Mahendran, Shilajit Ghosh, Daniel Gey Van Pittius, Udo Abah

**Affiliations:** 1 Cardiothoracic Surgery, Royal Stoke Hospital, Stoke-on-Trent, GBR; 2 Medical Education, Keele University, Stoke-on-Trent, GBR; 3 Pathology, Royal Stoke Hospital, Stoke-on-Trent, GBR

**Keywords:** brain metastases，lung cancer, extracranial meningioma, meningioma histopathology, primary pulmonary meningioma, recurrent meningioma

## Abstract

Extracranial metastatic meningiomas are an extremely rare type of metastatic meningiomas. They are often asymptomatic and found incidentally on imaging. In this report, we discuss a young lady who presented with atypical chest pains, in whom initial work-up demonstrated a lesion in the left lung which was subsequently surgically resected. There was some confusion regarding whether the lesion was a primary pulmonary meningioma or a metastasising meningioma, both of which present similarly on histology with spindle or oval-shaped cells arranged in whorls. Further analysis revealed that this lesion belonged to a rare subgroup called ‘benign metastasising meningioma’, where the histology does not show the characteristic features of a metastatic lesion (such as high mitotic rate or evidence of necrosis). In fact, this lesion was found to have no necrosis, patternless growth, nuclear atypia or lymphovascular invasion and minimal mitoses occurring rarely despite metastasising from the meninges. This finding triggered further investigations in the form of brain imaging, which identified recurrence of a left central meningioma (a slow-growing tumour arising from the meninges on the left side of the brain), which was successfully treated 11 years previously. Neurosurgical intervention is currently being planned. Pulmonary meningiomas are extremely rare. In a patient with a history of a central nervous system meningioma, a pulmonary meningioma should be investigated further to assess whether the lesion is a primary or secondary metastasis.

## Introduction

Meningiomas are the most common type of brain tumour, accounting for approximately 37.6% of all primary central nervous system (CNS) tumours [1]. They are lesions arising from the protective layer surrounding the brain, the meninges [2]. They are often classified based on the World Health Organization criteria of meningiomas into grades 1-3: benign, atypical, and malignant, respectively [3]. Those that are benign are generally characterised as non-cancerous and slow-growing tumours with symptoms only occurring if the lesion compresses a nerve [4]. Malignant meningiomas are extremely rare, accounting for only 1% of all meningiomas [5]. They often metastasise to areas outside the CNS and present with headaches, weakness, and seizures [6]. Here, we present a case of a benign meningioma which metastasised to the lungs without malignant transformation.

## Case presentation

A 39-year-old lady presented with atypical chest pains to her General Practitioner. Her past medical history was limited to a left clival meningioma (tumour affecting the meninges of the brain on the left side of the clivus of the skull) with a World Health Organization grading of 1 [2], for which she was treated with gamma knife radiosurgery 11 years ago. Five-year follow-up imaging showed initial shrinkage, then a stable appearance of the benign lesion.

**Figure 1:**
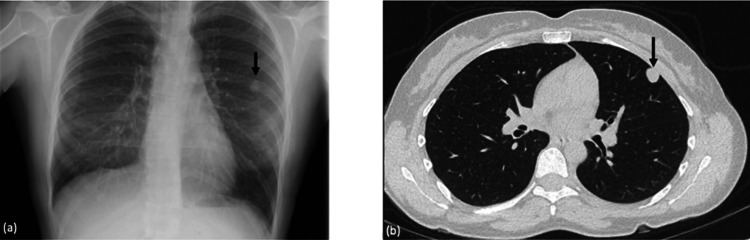
Initial imaging on investigation of a left-sided lung nodule (a) Chest x-ray showing a well-rounded nodule in the periphery of the left midzone measuring up to 1.5 cm. (b) CT thorax showing a solitary 14 mm subpleural nodule anteriorly in the lingula. Nodule is hyperdense (mean density 30-50 Hounsfield Unit (HU)) with probable cystic nature. Acute angulation with the costal surface suggests pulmonary rather than pleural origin.

A chest X-ray was requested, which showed a left-sided lung nodule (Figure 1a). This was further investigated with a CT scan, which showed a 14 mm lingula nodule (tongue-shaped projection from the upper lobe of the left lung) (Figure 1b). A half-body (neck to pelvis) Positron Emission Tomography (PET) scan showed the nodule was fluorodeoxyglucose (FDG) avid (high metabolic rate of the cells present, indicating tumour growth) but had no evidence of metastases. An attempt at a CT-guided biopsy was made, but it showed inconclusive results. She was therefore referred to the thoracic surgery team at our tertiary hospital for resection of the nodule. She had a left-sided video-assisted thoracic surgery where a left upper lobe wedge resection of the lesion was done.

Macroscopically, the lung wedge measured 40 x 19 x 18 mm. The solid white nodule within this section of lung measured 14 x 12 x 12 mm, abutting the pleura and lying 5 mm from the nearest stapled margin. Microscopically, the tumour was clear of the stapled margin by at least 5 mm and revealed a well-circumscribed neoplasm composed of solid sheets of medium to large cells with abundant pale eosinophilic cytoplasm with mild nuclear atypia. These cells formed syncytial whorls with concentric onion-bulb-like arrangement of cell membranes mixed with more rhabdoid-type cells forming clusters and containing moderately dense eccentrically placed eosinophilic cytoplasm.

An extensive panel of markers shows diffuse expression of vimentin in tumour cells with focal expression of epithelial membrane antigen (EMA), p63 (transcription factor of keratinocyte proliferation and embryonic epidermal growth) and CD56 (marker of natural killer cells and lymphomas). The tumour was negative for: cytokeratin CAM 5.2 (marker which is positive in adenocarcinomas and glandular epithelia), cytokeratin MNF 116 (marker for micrometastases in lymph nodes), cytokeratin AE1/AE3 (marker to rule out epithelial tumours or occult tumour cells of carcimoas in lymph nodes), SALL4 (staining for identification of germ cell tumours or hepatocellular carcinomas), Sox-10 (melanoma marker), S100 (melanoma marker), HMB45 (melanoma marker), thyroid transcription factor-1 (TTF1, distinguishes primary and metastatic lung carcinoma from non-lung primary tumours), synaptophysin (marker of neuroendocrine differentiation), chromogranin (neuroendocrine marker), estrogen receptor (ER, nuclear staining to test for breast lesions), GATA-3 (nuclear stain to differentiate mesothelioma from pulmonary adenocarcinoma), CDX-2 (marker to identify gastrointestinal origin for adenocarcinomas), actin (membranous or cytoplasmic staining to identify myoepithelial cells), CD34 (membranous stain to determine microvessel density), D2-40 (membranous staining to determine presence of lymphatics), CD99 (membranous staining to rule out Ewing sarcoma), CD117 and DOG-1 (cytoplasmic stainings to determine presence of gastrointestinal stromal tumours), and PAX-8 (nuclear stain differentiating primary pulmonary carcinomas from renal, Müllerian and thyroid metastatic carcinomas). The Ki-67/MIB-1 (nuclear staining, higher expression is proportional to the mitotic rate) proliferative index was very low at less than 5%.

**Figure 2:**
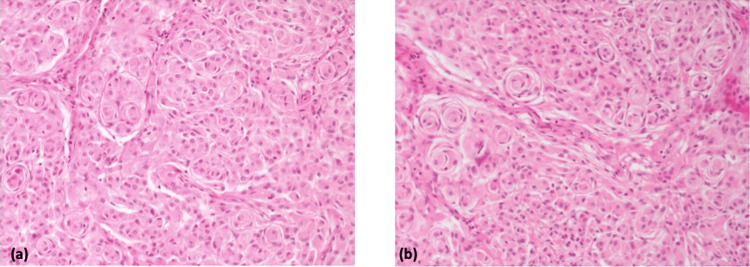
Histological images of benign metastatic meningioma on H&E staining (a-b) Distinctive morphology is seen in these slides with evident meningothelial whorls and rhabdoid cells.

The microscopic appearances of the lung lesion were those of low-grade epithelioid neoplasm with a very distinctive morphology with meningothelial whorls and rhabdoid cells (Figure 2). There was no evidence of necrosis, disordered growth, cellular crowding, nuclear/nucleolar atypia or lymphovascular invasion. Mitoses occurred rarely (up to 1 mitosis identified in 10 high-power fields) despite metastasising from the meninges. At first, the overall appearances favoured a primary pulmonary meningioma. However, a metastatic meningioma was another possibility that was considered, given the patient’s history. The histology was sent externally for further expert review, who advised that whilst meningiomas can be primary pulmonary neoplasms, they are more commonly metastatic, even when histology appears morphologically "indolent/low-grade/benign". Indeed, such lesions have been called "benign metastasising meningioma" by some. Given the clinical history, this appears to be the most likely scenario.

**Figure 3:**
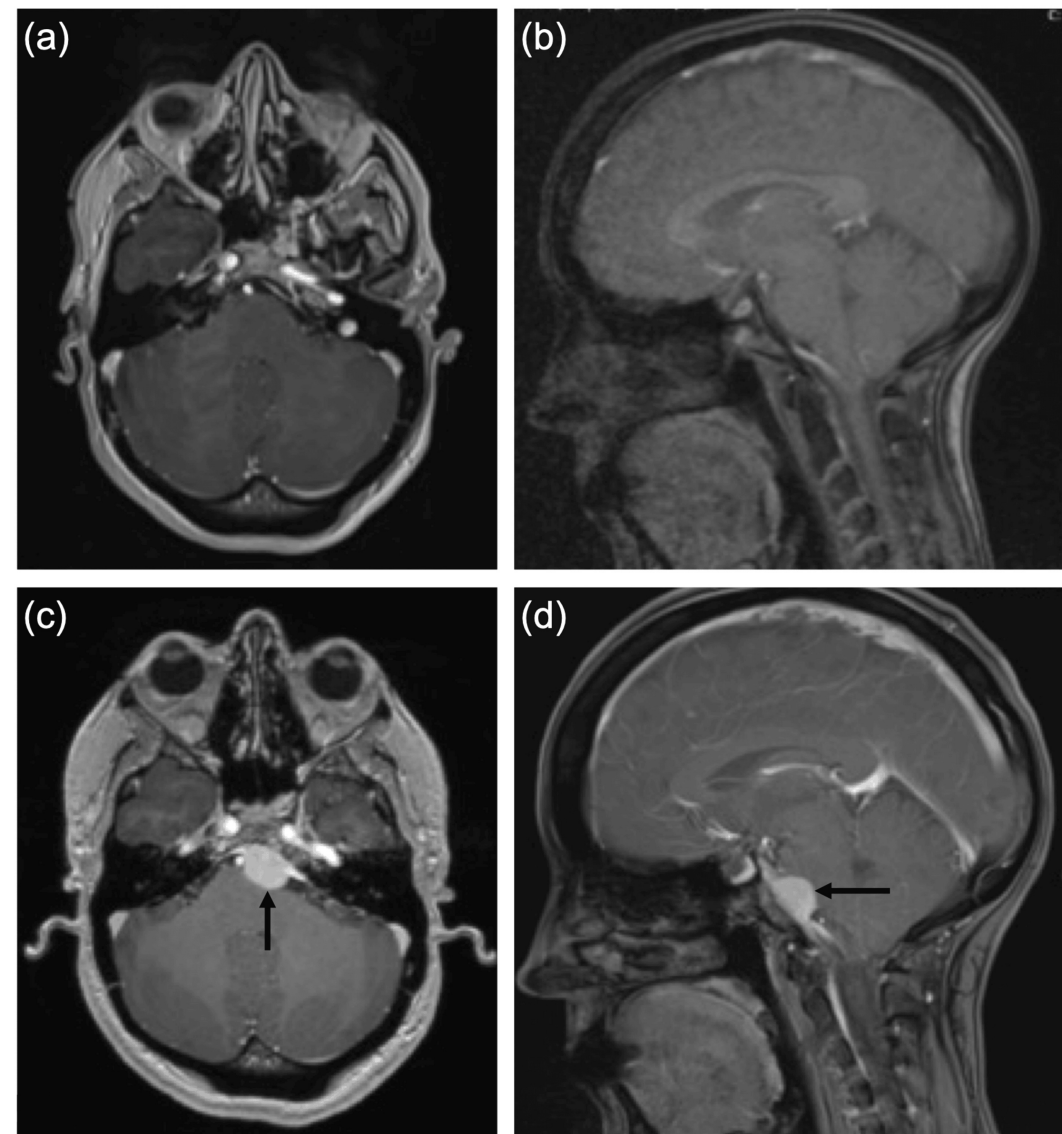
Recurrence of meningioma (a-b) 5-year magnetic resonance imaging (MRI) following gamma knife radiosurgery shows stable appearance of a left clival meningioma. (c-d) Recurrence of clival left central meningioma 11 years after initial diagnosis. Tumour measures 15 mm in depth, displacing the basilar artery to the right and indents the anterior pons without oedema. No extension into the Meckel’s cave or left internal auditory meatus noted.

This surprising finding prompted the patient to be re-referred to the neurosurgical team, who conducted repeat MRI brain imaging, last done 9 years previously, despite not having any neurological symptoms (Figure 3). This showed that the patient had a recurrence of the clival left central meningioma. Due to the previous radiotherapy, neurosurgical intervention is currently being planned, as further radiosurgery may do more harm.

## Discussion

Extracranial metastases from primary CNS meningiomas are extremely rare, accounting for less than 1% of all meningiomas [7]. In cases where meningiomas do metastasise outside the cranium, the most common site is the lung [8], followed by the liver, lymph nodes, and bone.

Metastasising meningioma and primary pulmonary meningioma cannot be differentiated histologically, particularly if they are low-grade, as in the case presented. Diagnosis is dependent on patient history and clinical investigations, specifically imaging of the CNS. Metastasising meningiomas may have the histological appearance of a low-grade, benign lesion as indicated by the Ki-67 proliferation index. The lesion itself, although having metastasised, may be benign - a contradiction indeed! These tumours are therefore called "benign metastasising meningiomas" and have been documented in the literature as being extremely rare, with only 70 cases reported since 1886 [8-10].

Given that the initial cranial tumour was a benign meningioma which had successfully undergone gamma knife radiosurgery, the possibility of a benign metastatic meningioma was considered less likely, given the benign features of the pulmonary nodule, including low-grade epithelioid cells, organised growth, and minimal mitoses, suggesting this lesion had not metastasised from the brain, as it did not show any malignant features characteristic of a malignant tumour. As a result, this raised the possibility of a primary pulmonary meningioma.

Primary pulmonary meningiomas are also sporadic, with less than 100 cases reported to date [11-13]. They are commonly asymptomatic and found incidentally. However, some cases have reported symptoms such as chest pain and breathlessness [11,13]. They may present as a solitary, slow-growing nodule in the lung and confer a good prognosis [11].

In this case, the pulmonary lesion was fully resected. However, long-term outcomes (including mortality and recurrence) of pulmonary metastasectomy for metastatic meningiomas are unknown and require further research.

## Conclusions

In conclusion, this case highlights that if a patient presents with a solitary lung nodule on a background of a cranial meningioma, one must consider the possibility of a metastatic meningioma. Although rare, benign metastatic meningioma, despite its paradoxical histological presentation and naming, must be considered if a lesion shows histological appearances of a meningioma. This tumour type does not follow the predictable nature of a benign cranial lesion, which, by definition, does not develop metastatic lesions. The importance of a thorough history and up-to-date imaging cannot be overstated, which eventually led to the final diagnosis of an extremely rare benign metastasising meningioma in this patient.
